# Beneficial effects of Cripto-1 for transarterial chemoembolization in hepatocellular carcinoma

**DOI:** 10.18632/aging.101951

**Published:** 2019-05-27

**Authors:** Xiao-shan Li, Jia-hong Wang, Xian-zi Yang, Lei Ma, Yan-xia Shi, Ye Song, Peng Jiang, Sha Gao, Ye Dong, Jin-rong Lin, Chuan Jin

**Affiliations:** 1 Department of Medical Oncology, Affiliated Cancer Hospital and Institute of Guangzhou Medical University, Guangzhou, Guangdong 510095, China; 2 Department of Abdominal Surgery, Affiliated Cancer Hospital and Institute of Guangzhou Medical University, Guangzhou, Guangdong 510095, China; 3 Senior coauthors; * Equal contribution

**Keywords:** hepatocellular carcinoma, Cripto-1, adjuvant TACE, prognosis

## Abstract

Cripto-1 may act as an independent predictor for prognosis in hepatocellular carcinoma (HCC). However, the function of Cripto-1 in HCC cells and its response to postoperative transarterial chemoembolization (TACE) in HCC patients remains unclearly. Up-regulated Cripto-1 expression boosted the ability of cell proliferation, migration and invasion in HCC cells *in vitro*. While opposite results were observed in HCC cells with down-regulated Cripto-1 expression. Cripto-1 expression was correlated with epithelial-mesenchymal transition (EMT) relevant biomarkers. Furthermore, in high Cripto-1 expression patients, those with adjuvant TACE had favorable TTR and OS times. On contrary, adjuvant TACE may promote tumor recurrence but had no influence on OS time in patients with low Cripto-1 expression. In different subgroups of vascular invasion, larger tumor size or liver cirrhosis, patients with adjuvant TACE had longer TTR and OS times than those without TACE in patients with high Cripto-1 expression, while they could not obtain benefits from adjuvant TACE in patients with low-expressed Cripto-1 expression. In conclusion, Cripto-1 may be a potential prognostic factor in predicting outcome of HCC patients with TACE therapy, and combined with Cripto-1 and tumor features may be helpful to stratify patients with respect to prognosis and response to adjuvant TACE.

## INTRODUCTION

Hepatocellular carcinoma (HCC) is one of the most common malignant tumors and a leading cause of cancer-related death [1, 2]. The prognosis of HCC patients remains unsatisfactory because of high incidence of recurrence after hepatectomy [3, 4]. And intrahepatic metastasis was the main types of recurrence after surgery [5]. It has been known that transcatheter arterial chemoembolization (TACE) is the most common method to prevent relapse and improve survival of HCC patients after surgery. It also reported that only selection patients could obtain benefits from adjuvant TACE [6]. Previous studies reported that patients with large tumors or venous invasion were suggested to accept adjuvant TACE 1-2 months after resection [6–8]. However, TACE makes damage to liver function, which may negatively influence the patients’ survival [9]. Moreover, prognosis may be very different in HCC patients with the same clinicopathologic features, which may due to the heterogeneity of biological behavior of tumor cells [10, 11]. Therefore, screening new predictive factors are important to identify individuals most likely to benefit from TACE, and is the major objective of personalized medicine [12].

Cripto-1 is a member of the EGF-CFC protein family, which contains an epidermal growth factor (EGF)-like domain and a cysteine-rich region called the Cripto/FRL1/Cryptic (CFC) domain (Cripto in humans, FRL1 in Xenopus, and Cryptic in mice) [13]. Cripto-1 could be used as a critical EMT inducer during embryonic development which is often reactivated in tumor cells, regulating the multistep progression of tumorigenesis [14]. Our previous study evaluated the clinical significance of Cripto-1 expression in HCC patients and revealed that Cripto-1 overexpression contributed to aggressiveness and poor prognosis of HCC patients [15]. Therefore, we hypothesized that the efficacy of postoperative adjuvant TACE on survival may be different in HCC patients with different Cripto-1 expression. In this study, we investigated the biological function of Cripto-1 in HCC cells and its response to postoperative adjuvant TACE in HCC patients.

## RESULTS

### Upregulated Cripto-1 expression promoted HCC cell proliferation and invasion

**Figure 1:**
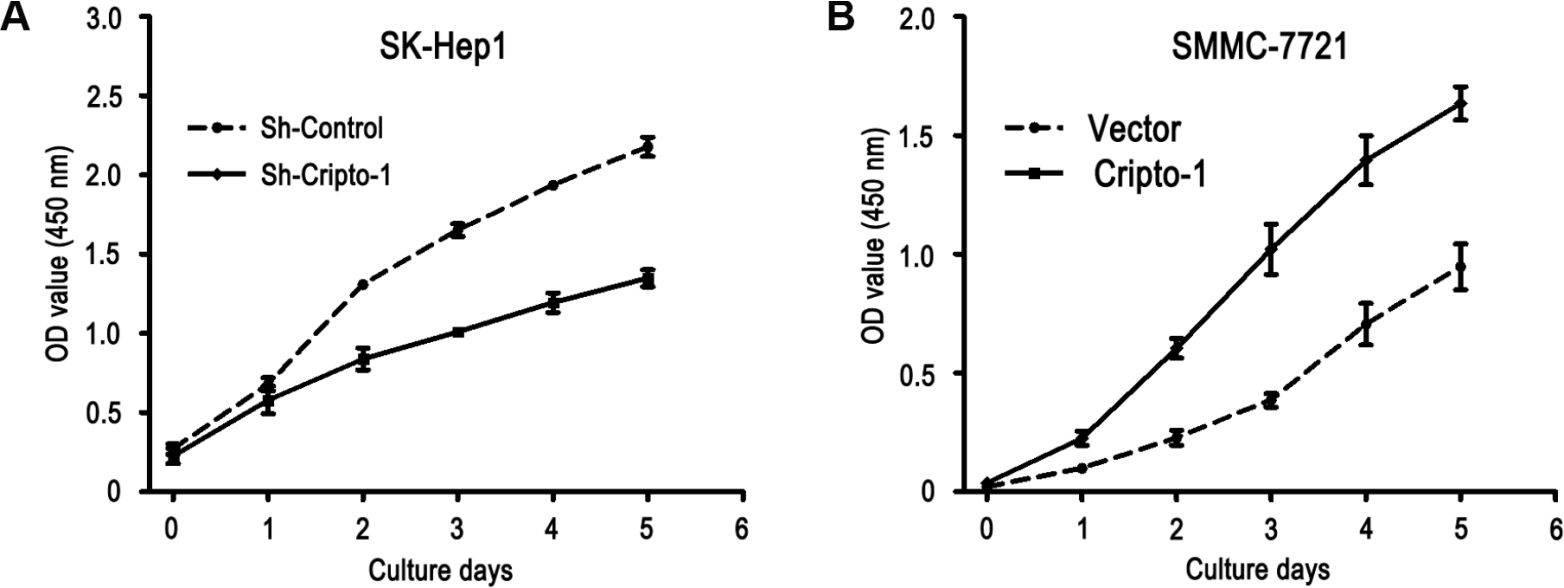
**Cripto-1 promotes cell proliferation in HCC cells.** The cell viability was inhibited in Cripto-1-silenced cells compared with the control cells (**A**), while the opposite effect of cell viability was observed in Cripto-1-transfected cells (**B**).

**Figure 2:**
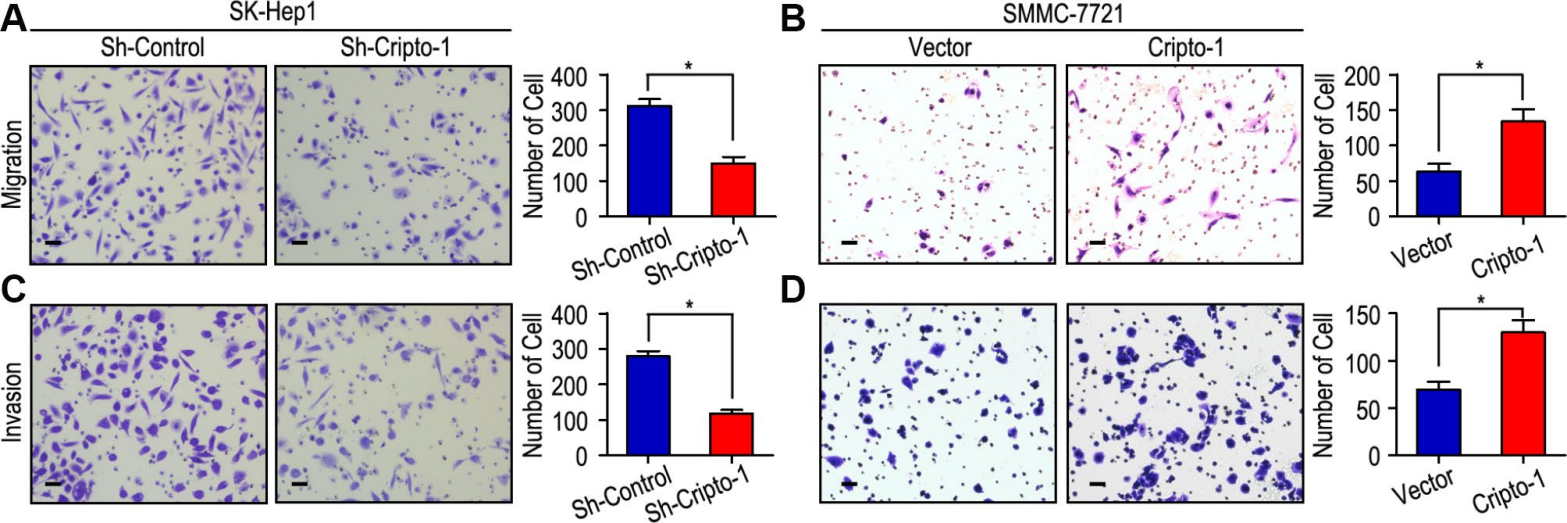
**Cripto-1 promotes cell migration and invasion in HCC cell lines.** (**A**, **C**) Down-regulated Cripto-1 expression in SK-Hep1 cells and suppressed cell migration and invasion. (**B**, **D**) Up-regulated Cripto-1 expression in SMMC-7721 cells and accelerated cell migration and invasion. The scale bar represents 50 μm.

Our previous study had showed that Cripto-1 could be used as a potential prognostic factor for the survival of HCC patients. We further explored the function of Cripto-1 in tumor cell proliferation, migration and invasion in HCC cells. The CCK-8 assay results showed that down-regulated Cripto-1 expression may inhibit tumor cells proliferation in SK-Hep1 cells, while opposite results were showed in up-regulated Cripto-1 expression of SMMC-7721 cells (Figure 1). Transwell invasion assay found that lower invasion and migration ability was observed in sh-Cripto-1-SK-Hep1 cells, compared with those with shControl-SK-Hep1 cells (both P < 0.001, Figure 2A, 2C), while the ability of cell motility and invasion was increased in Cripto-1-SMMC-7721 cells, compared with those with Vector-SMMC-7721 cells (both P < 0.001, Figure 2B, 2D).

**Figure 3:**
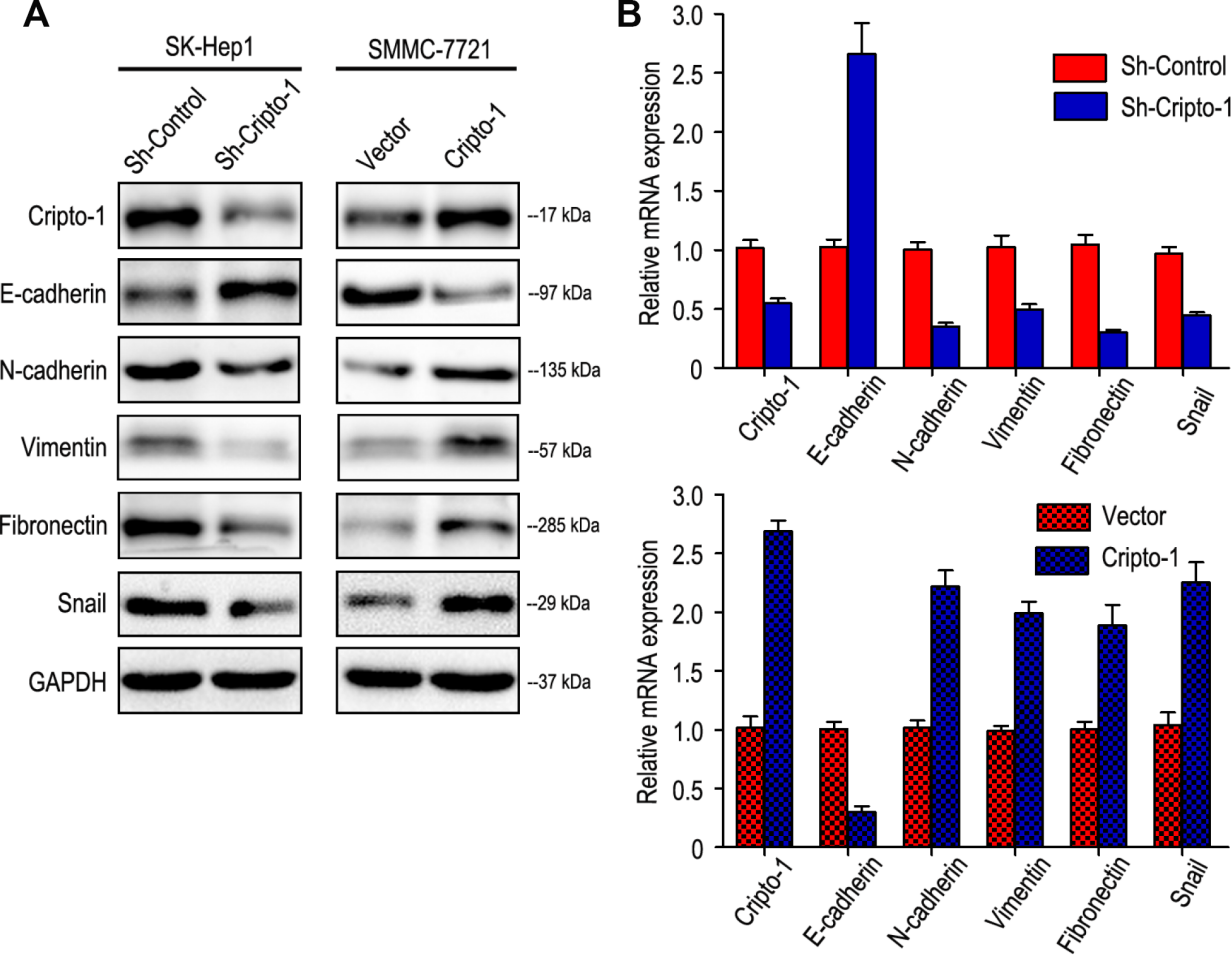
**Cripto-1 expression was correlated with the expression of epithelial-mesenchymal transition (EMT) relevant markers.** Western blot (**A**) and RT-PCR (**B**) results showed that transfecting siRNA against Cripto-1 decreased Cripto-1 expression, down-regulated N-cadherin, Vimentin, Fibronectin and Snail and up-regulated E-cadherin. While opposite results were showed in HCC cells with up-regulated Cripto-1 expression.

As Epithelial-mesenchymal transition (EMT) is a key process in cancer metastasis by which tumor cells acquire migratory characteristics, thereby disassociating from the primary tumor and migrating to distant sites [16, 17], the effect of Cripto-1 on EMT was examined by investigating the expression levels of EMT relevant markers. Western blot and RT-PCR results revealed that increased expression of E-cadherin with decreased expression of N-cadherin, Vimentin, Fibronectin and Snail appeared in sh-Cripto-1-SK-Hep1 cells, compared with the control cells (Figure 3A and 3B). An opposite expression pattern of these genes was observed in Cripto-1-SMMC-7721 cells (Figure 3A and 3B).

### Prognostic significance of postoperative adjuvant TACE in different Cripto-1 expression

**Figure 4:**
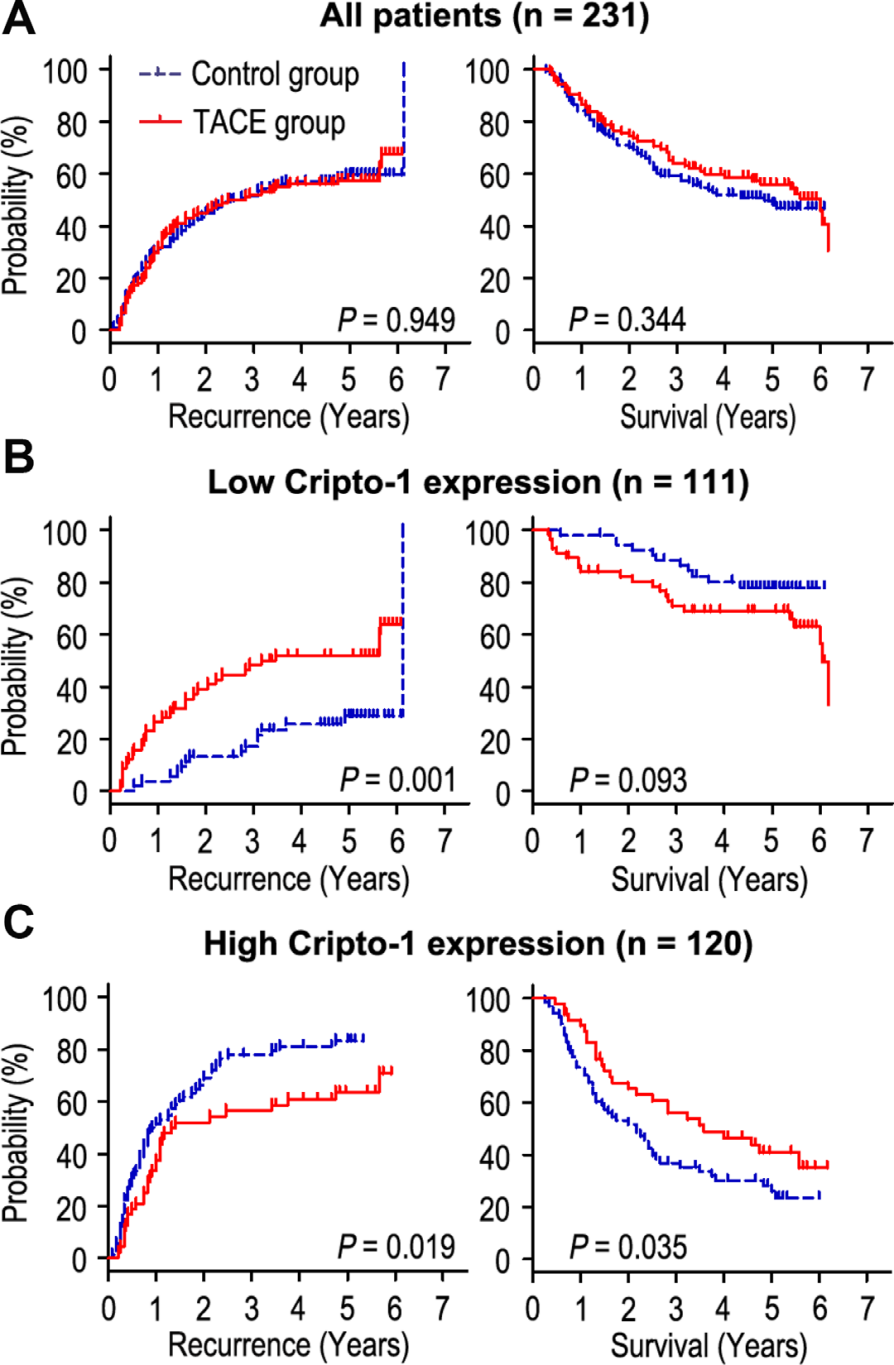
**Prognostic significance of adjuvant TACE in HCC patients according to Cripto-1 expression.** Adjuvant TACE could not increase the OS or TTR times in all HCC patients (**A**). Kaplan-Meier analysis of the association between adjuvant TACE therapy and OS/TTR in patients with low Cripto-1 expression (**B**) and high Cripto-1 expression (**C**).

Adjuvant TACE is one of the most common methods to prevent tumor relapse and prolong survival time in HCC. However, the curative effect of adjuvant TACE remains controversial. In this study, our results revealed that adjuvant TACE after hepatectomy could not increase the OS or TTR times in the whole study population (Figure 4A). As Cripto-1 plays an important role in tumor progression and could be an independent prognostic biomarker for HCC patients, we further explored whether the response of patients to adjuvant TACE therapy was different in different Cripto-1 expression of HCC patients. Interestingly, adjuvant TACE may promote tumor relapse and had no influence on OS time in patients with low Cripto-1 expression (Figure 4B). On the contrary, in high Cripto-1 expression patients, those with adjuvant TACE had favorable TTR and OS times (Figure 4C). In patients with high Cripto-1 expression, the median of TTR was 13.0 months, while the median of OS was 26.5 months. The median of TTR and OS times for adjuvant TACE group were 16.5 months and 36.5 months, while there were 10.5 months and 22.5 months in control group. Furthermore, the 5-year TTR and OS rates of the adjuvant TACE group were 36.3% and 40.9%, which were significantly higher than that of the control group (16.7% and 28.1%) (Figure 4C).

Cox proportional-hazards regression was used to analyze the correlation of Cripto-1 expression with response to adjuvant TACE in HCC patients. Our data revealed that adjuvant TACE could not be an independent prognostic factor for TTR or OS in HCC patients with low Cripto-1 expression (Table 1). Interestingly, in patients with high Cripto-1 expression, the results showed that without received TACE (*P* = 0.019), larger tumor size (*P* = 0.01), multiple tumor (*P* = 0.001), satellite nodule (*P* = 0.025) and vascular invasion (*P* = 0.005) were unfavourable factors for TTR in HCC patients. Moreover, Kaplan-Meier analysis revealed that without received TACE (*P* = 0.035), high AFP level (*P* = 0.030), larger tumor size (*P* = 0.002), multiple tumor (*P* < 0.001), satellite nodule (*P* = 0.007) and vascular invasion (*P* < 0.001) were positively associated with shorter OS in HCC patients (Table 2). Multivariate Cox regression analysis showed that patients who received adjuvant TACE had longer TTR times than those without received TACE therapy (HR = 0.371, 95% CI = 0.229-0.602, *P* < 0.001). In addition, adjuvant TACE was a potential prognostic biomarker for OS (HR = 0.280, 95% CI = 0.162-0.484, *P* < 0.001) in patients with high Cripto-1 expression (Table 2).

**Table 1:** Univariate and multivariate analysis of prognosis in low Cripto-1 expression of HCC patients

**Variables**	**TTR**				**OS**			
	**Univariate**	**Multivariate**			**Univariate**	**Multivariate**		
	***P*-value**	***P*-value**	**HR**	**95% CI**	***P*-value**	***P*-value**	**HR**	**95% CI**
Gender (Female vs. Male)	NS	NS			NS	NS		
Age, years (≤ 50 vs. > 50)	NS	NS			NS	NS		
AFP (ng/mL) (≤ 400 vs. > 400)	NS	NS			NS	NS		
HBsAg (Negative vs. Positive)	NS	NS			NS	NS		
GGT (U/l) (≤ 50 vs. > 50)	NS	NS			NS	NS		
Liver cirrhosis (No vs. Yes)	0.042	NS			0.027	0.024	3.052	1.157–8.053
Tumor size (cm) (≤ 5 vs. > 5)	0.010	NS			0.036	NS		
Tumor number (Single vs. Multiple)	0.006	NS			NS	NS		
Satellite nodule (No vs. Yes)	0.012	NS			NS	NS		
Tumor capsule (No/ incomplete vs. Complete)	NS	NS			NS	NS		
Tumor differentiation (I–II vs. III–IV)	NS	NS			NS	NS		
Vascular invasion (No vs. Yes)	0.006	NS			0.016	NS		
Adjuvant TACE (No vs. Yes)	0.001	NS			NS	NS		

TTR time to recurrence, GGT gamma-glutamyltransferase, AFP α-fetoprotein, TACE transcatheter arterial chemoembolization, NS not significant, HR hazard ratio, CI confidential interval.

**Table 2:** Univariate and multivariate analysis of prognosis in high Cripto-1 expression of HCC patients

**Variables**	**TTR**				**OS**			
	**Univariate**	**Multivariate**			**Univariate**	**Multivariate**		
	***P*-value**	***P*-value**	**HR**	**95% CI**	***P*-value**	***P*-value**	**HR**	**95% CI**
Gender (Female vs. Male)	NS	NS			NS	NS		
Age, years (≤ 50 vs. > 50)	NS	NS			NS	NS		
AFP (ng/mL) (≤ 400 vs. > 400)	NS	NS			0.030	0.017	1.785	1.111–2.867
HBsAg (Negative vs. Positive)	NS	NS			NS	NS		
GGT (U/l) (≤ 50 vs. > 50)	NS	NS			NS	NS		
Liver cirrhosis (No vs. Yes)	NS	NS			NS	NS		
Tumor size (cm) (≤ 5 vs. > 5)	0.010	NS			0.002	NS		
Tumor number (Single vs. Multiple)	0.001	< 0.001	2.982	1.686–5.276	< 0.001	< 0.001	5.348	2.772–10.316
Satellite nodule (No vs. Yes)	0.025	NS			0.007	NS		
Tumor capsule (No/ incomplete vs. Complete)	NS	NS			NS	NS		
Tumor differentiation (I-II vs. III-IV)	NS	NS			NS	NS		
Vascular invasion (No vs. Yes)	0.005	0.004	2.142	1.271–3.612	< 0.001	0.002	2.399	1.380–4.168
Adjuvant TACE (No vs. Yes)	0.019	< 0.001	0.371	0.229–0.602	0.035	< 0.001	0.280	0.162–0.484

TTR time to recurrence, GGT gamma-glutamyltransferase, AFP α-fetoprotein, TACE transcatheter arterial chemoembolization, NS not significant, HR hazard ratio, CI confidential interval.

### Cripto-1 expression predicts response of adjuvant TACE in clinical subgroups

**Figure 5:**
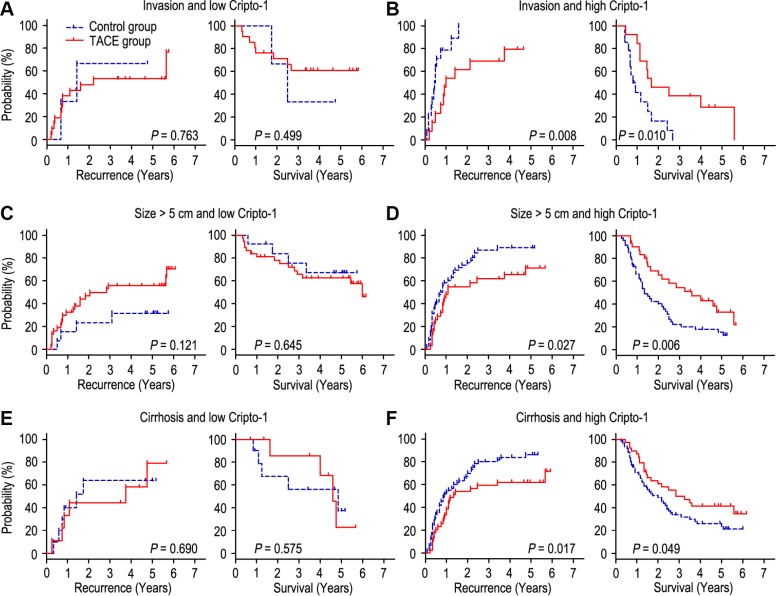
**combination of Cripto-1 and tumor features predicts response to postoperative TACE**. All patients were divided according to Cripto-1 levels within vascular invasion (**A**, **B**), lager tumor size (**C**, **D**) or liver cirrhosis (**E**, **F**). Kaplan-Meier survival estimates and log-rank tests were used to analyze the correlation of adjuvant TACE therapy and OS/TTR in different subgroups.

The HCC patients with vascular invasion or larger tumor (> 5 cm in diameter) are recommended to receive TACE therapy 1-2 months after hepatectomy [6–8]. It has been known that liver cirrhosis is a significant risk factor for post-operative recurrence [18, 19]. Our findings had similar results with previous study. We found that adjuvant TACE could reduce the recurrence rate and prolong the OS time in HCC patients with vascular invasion (Supplementary Figure 1B) and larger tumor (Supplementary Figure 1D), while adjuvant TACE had no effect on patients with no vascular invasion (Supplementary Figure 1A), small tumor (Supplementary Figure 1C), liver cirrhosis (Supplementary Figure 1F). Interestingly, adjuvant TACE may even promote tumor recurrence in patients with no liver cirrhosis (Supplementary Figure 1E). Furthermore, we wonder whether all the patients with vascular invasion or larger tumor could obtain a benefit from adjuvant TACE after surgery, and whether all the liver cirrhosis patients could not have a benefit from adjuvant TACE. We further assessed the discriminative power of Cripto-1 expression on postoperative adjuvant TACE in patients with vascular invasion, large tumor and liver cirrhosis. In the patients with vascular invasion subgroup (n = 51), low-expressed Cripto-1 patients had no response on adjuvant TACE (Figure 5A), On the contrary, of the patients with high Cripto-1 expression, adjuvant TACE had a better median TTR and OS times than the control group (median TTR for adjuvant TACE [n = 13] versus control group [n = 14]: 12.0 months versus 5.5 months, *P* = 0.008; median OS for adjuvant TACE [n = 13] versus control group [n = 14]: 20.0 months versus 9.5 months, *P* = 0.010; Figure 5B). In the patients with larger tumor subgroup (n = 129), patient with low Cripto-1 expression could not have a benefit from adjuvant TACE (Figure 5C). Contrarily, adjuvant TACE could prolong the TTR and OS of patients with high Cripto-1 expression (median TTR for adjuvant TACE [n = 31] versus control group [n = 48]: 13.0 months versus 10.0 months, *P* = 0.027; median OS for adjuvant TACE [n = 31] versus control group [n = 48]: 34.0 months versus 15.5 months, *P* = 0.006; Figure 5D). In the patients with liver cirrhosis subgroup (n = 179), adjuvant TACE had no effect on patients with low Cripto-1 expression (Figure 5E). Interestingly, adjuvant TACE was associated with significant improvement in TTR and OS of patients with high Cripto-1 expression (median TTR for adjuvant TACE [n = 39] versus control group [n = 62]: 16.0 months versus 10.0 months, *P* = 0.017; median OS for adjuvant TACE [n = 39] versus control group [n = 62]: 34.0 months versus 20.5 months, *P* = 0.049; Figure 5F).

## DISCUSSION

Adjuvant TACE is one of the most common methods to prevent recurrence after hepatectomy. However, the efficacy of TACE remains controversial. Some studies reported that adjuvant TACE could inhibit tumor relapse and improve prognosis of patients, while others found that the patients with resectable HCC could not obtain benefits from adjuvant TACE [20–23]. Moreover, according to liver function damage and expense caused by TACE therapy, it is necessary to select the appropriate patients who may have benefits from TACE after surgery. It has been reported that identification of potential biomarkers for predicting response to adjuvant TACE is important to improve the curative effect of TACE. In the present study, we evaluated the function of Cripto-1 in HCC cells, and investigated the clinical effect of Cripto-1 in predicting the response of adjuvant TACE in patients after hepatectomy.

Cripto-1 regulates important steps in early embryogenesis, and plays a pivotal role in stem cell maintenance and tumor progression. Abnormal expression of Cripto-1 has been detected and could be used as predictor for prognosis in many human cancers, including HCC [15, 24], glioblastoma [25], breast cancer [26], lung adenocarcinoma [27] and gastric cancer [28]. Our previous results showed that Cripto-1 overexpression was associated with larger tumor, TNM stage, BCLC stage and tumor recurrence, and Cripto-1 expression could be a prognostic factor for survival in HCC patients [15]. In this study, we found that up-regulated Cripto-1 expression could accelerate tumor cell proliferation, migration, and invasion in HCC cells *in vitro*. Moreover, opposite results could be shown when Cripto-1 was down-regulated. It has been reported that Cripto-1 had a pivotal role in development of EMT, and may be involved in the reprogramming of differentiated tumor cells into cancer stem cells vis inducing the EMT pathway [29]. Because EMT plays an important role in tumor invasion and metastasis during tumor progression, we further evaluated the associated of Cripto-1 expression with biomarkers of the EMT pathway and revealed that Cripto-1 expression was positively correlated with N-cadherin, Vimentin, Fibronectin and Snail expression and negatively correlated with E-cadherin expression in HCC cells. Furthermore, Lo RC et al. investigated the significance of Cripto-1 in HCC cells stemness and substantiated the role of Cripto-1 in determining stemness phenotypes of HCC by the ways of modulating the Wnt/β-catenin signaling cascade [24]. These results suggest that Cripto-1 enhances cell proliferation and invasion through induction of EMT and activation of Wnt/β-catenin signaling pathway in HCC.

To our knowledge, this is the first report investigating the influence of Cripto-1 protein expression in HCC patients undergoing TACE after surgery. Our data revealed that TACE could not increase the survival time (TTR and OS) of HCC patients in this whole study population, which was consistent with previous results [30]. Surprisingly, when the HCC patients were subdivided into 2 groups based on the Cripto-1 expression, the efficacy of adjuvant TACE in different subgroups were totally different. Our results showed that, in the patients with low Cripto-1 expression, adjuvant TACE may promote tumor recurrence and had no significant difference in OS, compared with those without received TACE. However, multivariate analysis showed that TACE therapy could not be used as an independent prognostic factor for patients with low Cripto-1 expression. On the contrary, the patients with high Cripto-1 expression had improved TTR and OS when treated with adjuvant TACE, and TACE therapy was a predictor biomarker for TTR and OS in these patients. These results indicate that Cripto-1 might act as an indicator for selecting HCC patients for TACE therapy.

Previous studies showed that patients with larger tumor size, venous invasion or liver cirrhosis who had high risks of relapse were suggested to treat with adjuvant TACE [31–34]. However, even the patients with similar clinicopathologic parameters may have different outcome when received adjuvant TACE after surgery [23, 35–37]. Therefore, we combined Cripto-1 protein expression with high risk factors of recurrence to evaluate the response of adjuvant TACE in HCC patients. In patients with vascular invasion, larger tumor size or liver cirrhosis, low-expressed Cripto-1 patients could not obtain benefits from adjuvant TACE, while the patients with adjuvant TACE had longer TTR and OS times than those without TACE in patients with high Cripto-1 expression. These results suggest that Cripto-1 could help identify different outcome in HCC patients with the same clinicopathological parameters who undergoing TACE after hepatectomy, thus offering a rationale for treatment according to a combination of genotype and tumor features of a patients.

## MATERIALS AND METHODS

### Patients and specimens

The informed consents were provided and experiment was approved by the Institutional Review Board and Human Ethics Committee of Affiliated Cancer Hospital & Institute of Guangzhou Medical University.

All the 231 HCC samples were collected from Department of Hepatobiliary Oncology, Sun Yat-sen University (Guangzhou, China) between July 2007 and January 2009 (Supplementary Table 1). Among these patients, 126 patients (54.5%) had undergone curative resections only, and 105 patients (45.5%) received adjuvant TACE 1–2 months after hepatectomy. The patients included in this study following the inclusion: (1) all the tissues were pathological diagnosis; (2) no distant metastases or other malignant diseases (based on enhanced CT and/or MRI); (3) no chemotherapy or radiotherapy before surgery. All patients were enrolled consecutively according to resection tissues and follow-up data. Tumor stage was classified base on the 7^th^ Edition TNM classification of the American Joint Committee on Cancer Staging and the Barcelona Clinic Liver Cancer (BCLC) staging system.

### Establishment of Cripto-1 knockdown HCC cells

In this study, we used two human HCC cell lines: SK-Hep1 and SMCC-7721. The two cell lines were obtained from the Liver Cancer Institute of Fudan University (Shanghai, China) and were maintained in Dulbecco’s Modified Eagle’s Medium (DMEM) containing 1% penicillin and streptomycin, supplemented with 10% fetal bovine serum (FBS).

Lentiviral containing short hairpin RNAs (shRNA) targeting Cripto-1 was purchased from Hanbio Biotechnology (Hanbio Biotechnology Co., Ltd.) and transfected into SK-Hep1 cell using Lipofectamine 2000 (Invitrogen) following manufacturer’s protocol. Cells transfected with empty vector were used as controls. Stable Cripto-1 knockdown expression clones were selected by Geneticin (Rache Diagnostics, Indianapolis, IN) at the concentration of 500ug/ml.

### Plasmid constructs and transfection

Human Cripto-1 cDNA was amplified by PCR and cloned into pcDNA3.1(+) expression vector (Invitrogen, Carlsbad, CA), and then transfected into SMMC-7721 cell using Lipofectamine 2000 (Invitrogen) following manufacturer’s protocol. Cell transfected with empty vector were used as controls. Stable Cripto-1-expressing clones were selected by Geneticin (Rache Diagnostics, Indianapolis, IN) at the concentration of 500ug/ml.

### Cell counting kit-8 assay

The HCC cells were detached with trypsin and seeded on a 96-well plate (1x10^3^ cells/well). After culture in DMEM culture medium supplemented with 10% FBS to allow cells become adherent, cells were observed at different time points (0 h, 24 h, 48 h, 72 h, 96 h and 120 h). Then 10 μl CCK-8 solution was added into each well at different time points and the plate was incubated for additional 2 h. And then, the absorbance at 450 nm was measured using a microplate reader. Wells without addition of CCK-8 solution were taken as the blank. The cell viability was calculated using the formula: Cell viability (100%) = (mean OD of experimental group - mean OD of blank group)/(mean OD of control group - mean OD of blank group) x 100. We performed 3 parallel repeats in each group.

### Western blotting

Cell lysates were subjected to 7.5–10% PAGE and transferred to nitrocellulose filter membranes. The membranes were blocked for 1 h in 5 % non-fat dry milk diluted with TBST (10 mM Tris–HCl and 0.05 % Tween 20). The membranes were incubated with the Cripto-1 rabbit polyclonal antibody (working dilution 1:200, Abcam, #ab19917, UK), E-cadherin mouse monoclonal antibody (working dilution 1:300, Abcam, #ab1416, UK), N-cadherin rabbit polyclonal antibody (working dilution 1:200, Abcam, #ab18203, UK), Vimentin mouse monoclonal antibody (working dilution 1:200, Abcam, #ab8978, UK), Fibronectin rabbit polyclonal antibody (working dilution 1:300, Abcam, #ab2413, UK), Snail rabbit polyclonal antibody (working dilution 1:200, Abcam, #ab82846, UK), GAPDH rabbit polyclonal antibody (working dilution 1:5000, Abcam, #ab181602, UK) overnight at 4°C, followed by incubation with appropriate secondary antibodies at room temperature for 2h. The membranes were washed with PBS for three times, and the immunoreactive bands were visualized using an ECL plus Kit, according to the manufacturer’s instructions.

### Transwell invasion assays

Invasion assay was performed with BD BioCoat Matrigel Invasion Chambers (Becton Dickinson Labware, Franklin Lakes, NJ) following the manufacturer’s instructions. The matrigel membrane was stained with crystal violet, and migrated cells were counted under a microscope.

### Total RNA extraction and qRT-PCR

Total RNA was extracted from HCC cell lines using TRIzol reagent (Invitrogen) following manufacturer’s introductions. The total RNA (2 µg) was reverse transcribed using a PrimeScript RT Kit (Takara, Dalian) for first-strand cDNA synthesis. The primer sequences were: Cripto-1, forward primer, 5′-GGGATACAGCACAGTAAGGAG-3′, reverse primer, 5′-ACGGTGGTAGTTCTGGAGTC-3′. GAPDH, forward primer, 5′-CTCCTCCTGTTCGACAGTCAGC-3′, reverse primer, 5′-CCCAATACGACCAAATCCGTT-3′. E-cadherin, forward primer, 5′-CGAGAGCTACACGTTCACGG-3′, reverse primer 5′-GGGTGTCGAGGGAAAAATAGG-3′. N-cadherin, forward primer, 5′-CACGCCGAGCCCCAGTAT-3′, reverse primer 5′-GCCCCCAGTCGTTCAGGTAA-3′. Vimentin, forward primer, 5′-GACGCCATCAACACCGAGTT-3′, reverse primer, 5′-CTTTGTCGTTGGTTAGCTGGT-3′. Fibronectin, forward primer, 5′-CAGTGGGAGACCTCGAGAAG-3′, reverse primer, 5′-TCCCTCGGAACATCAGAAAC-3′. Snail, forward primer, 5′-TTCTCTAGGCCCTGGCTGC-3′, reverse primer, 5′-TACTTCTGACATCTGAGTGGGTCTG-3′. Expression of Cripto-1 gene and EMT relevant genes were examined by Power SYBR^®^ Green PCR Master Mix (Applied Biosystems, Carlsbad, CA), and the assay was performed on an ABI PRISM 7900 Sequence Detector. The relative expression level (defined as fold change) of Cripto-1 (2^-△△Ct^) was normalized to the endogenous 18S rRNA reference (△Ct) and related to the amount of target gene in control sample, which was defined as the calibrator at 1.0.

### TACE treatment

All the patients had received adjuvant TACE 1-2 months after surgery. Hepatic arterial angiography was performed firstly, and then preventive chemoembolization was done among patients without tumor stain in the remaining liver. The regimen for preventive adjuvant TACE consisted of lobaplatin 50 mg, Epirubicin (EPI) 50 mg and lipiodol 5 ml. A Contrast-enhanced CT or MRI was performed one month later, and the regimen was finished.

### Follow-up

The last follow-up was October 2017. All the patients were followed up every 2 months in the first 2 years after first TACE treatment and every 3-6 months afterwards, with routine monitoring by serum α-fetoprotein (AFP) level, liver function tests, abdominal ultrasonography, and contrast-enhanced computed tomography or magnetic resonance imaging. The main causes of death were HCC recurrence or complicated cirrhosis of the liver.

### Statistical analysis

The SPSS software package (version 16.0; Chicago, IL, USA) was used for the statistical analysis. The experimental data between two groups were performed by a two-tailed Student’s *t*-test. The mRNA level of Cripto-1 and EMT relevant markers in HCC cells were compared using paired Student’s *t*-test. The Kaplan-Meier analysis with log-rank test was used to calculate the survival time of HCC patients. The Cox proportional hazard regression model was used to analyze the independent prognostic biomarkers for HCC patients. All *P* values were two-sided and a *P* value < 0.05 was considered as statistically significant.

## CONCLUSIONS

It has been reported that molecular diagnosis for guiding targeted therapies, such as KRAS and BRAF mutation test in colorectal cancer [38], HER-2 amplification test in breast cancer [39], and BRAF mutation test in melanoma [40], become more and more important in cancer management. However, biomarker-guided therapy for HCC patients is still not available in clinical treatment. Our results identified that Cripto-1 may act as a potential prognostic factor in predicting the outcome of HCC patients with TACE therapy, and combined with Cripto-1 expression and tumor features may be helpful to stratify patients with respect to prognosis and response to adjuvant TACE therapy.

## Supplementary Material

Supplementary Figure and Table
