# Supplementary material for: Beneficial effects of Cripto-1 for transarterial chemoembolization in hepatocellular carcinoma
Source: Aging (Albany NY). 2019 May 27;11(10):2998–3011. doi: 10.18632/aging.101951 (PMC6555445; doi:10.18632/aging.101951)
Supplement: Supplementary Figure and Table [file aging-11-101951-s001.pdf]

SUPPLEMENTARY FIGURES

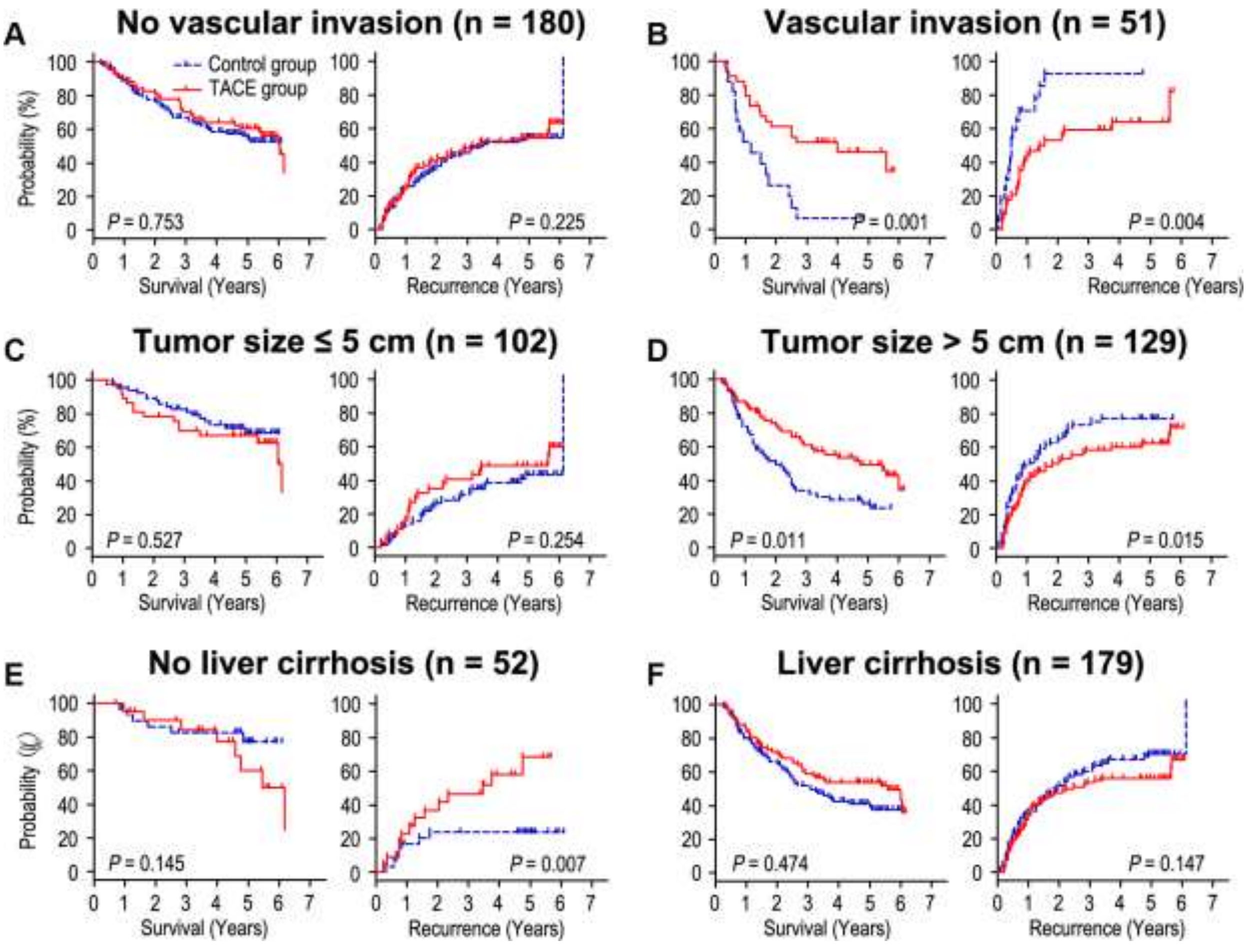

Supplementary Figure 1. Prognostic significance of postoperative adjuvant TACE in several clinical subgroups. All patients were stratified according to vascular invasion (**A**, **B**), tumor size (**C**, **D**) or liver cirrhosis (**E**, **F**). Kaplan-Meier survival estimates and log-rank tests were used to analyze the correlation of adjuvant TACE therapy and OS/TTR in clinical subgroups.

Supplementary Table 1. Patient characteristics.

Variable	No. of patients (%)
No. of patients	231 (100)
Age: Median [range], y	48 [15–80]
Gender	
Female	21 (9.1)
Male	210 (90.9)
HBsAg	
Negative	17 (7.4)
Positive	214 (92.6)
AFP: Median [range], ng/mL	174.1 [0.9–121000]
GGT: Median [range], U/l	55.4 [3.5–472.0]
Tumor size: Median [range], cm	6.0 [1.0–21.0]
Liver cirrhosis	
No	52 (22.5)
Yes	179 (77.5)
Child-Pugh class	
A	228 (98.7)
B	3 (1.3)
Tumor number	
Single	156 (67.5)
Multiple	75 (32.5)
Satellite nodule	
No	167 (72.3)
Yes	64 (27.7)
Tumor capsule	
No/incomplete	160 (69.3)
Complete	71 (30.7)
Tumor differentiation	
I	9 (3.9)
II	137 (59.3)
III	80 (34.6)
IV	5 (2.2)
Vascular invasion	
No	180 (77.9)
Yes	51 (22.1)
TNM stage	
I	114 (49.4)
II	28 (12.1)
III	89 (38.5)
BCLC stage	
0	14 (6.0)
A	63 (27.3)
B	100 (43.3)
C	54 (23.4)
Adjuvant TACE	
No	126 (54.5)
Yes	105 (45.5)
